# Why the growth of arboviral diseases necessitates a new generation of global risk maps and future projections

**DOI:** 10.1371/journal.pcbi.1012771

**Published:** 2025-04-04

**Authors:** Oliver J. Brady, Leonardo S. Bastos, Jamie M. Caldwell, Simon Cauchemez, Hannah E. Clapham, Illaria Dorigatti, Katy A. M. Gaythorpe, Wenbiao Hu, Laith Hussain-Alkhateeb, Michael A. Johansson, Ahyoung Lim, Velma K. Lopez, Richard James Maude, Jane P. Messina, Erin A. Mordecai, Andrew Townsend Peterson, Isabel Rodriquez-Barraquer, Ingrid B. Rabe, Diana P. Rojas, Sadie J. Ryan, Henrik Salje, Jan C. Semenza, Quan Minh Tran

**Affiliations:** 1 Department of Infectious Disease Epidemiology and Dynamics, London School of Hygiene and Tropical Medicine, London, United Kingdom; 2 Centre for Mathematical Modelling of Infectious Diseases, London School of Hygiene and Tropical Medicine, London, United Kingdom; 3 Centre on Climate Change and Planetary Health, London School of Hygiene and Tropical Medicine, London, United Kingdom; 4 Scientific Computing Programme, Oswaldo Cruz Foundation: Fundacao Oswaldo Cruz, Rio de Janeiro, Brazil; 5 High Meadows Environmental Institute, Princeton University, Princeton, New Jersey, United States of America; 6 Mathematical Modelling of Infectious Diseases Unit, Institut Pasteur, Université Paris Cité, UMR2000 CNRS, Paris, France; 7 Saw Swee Hock School of Public Health, National University of Singapore, Singapore, Singapore; 8 Medical Research Council Centre for Global Infectious Disease Analysis, Imperial College London, London, United Kingdom; 9 School of Public Health and Social Work, Queensland University of Technology, Brisbane, Australia; 10 Global Health Research Group, School of Public Health and Community Medicine, University of Gothenburg: Goteborgs Universitet, Gothenburg, Sweden; 11 Population Health Research Section, King Abdullah International Medical Research Center (KAIMRC), Riyadh, Saudi Arabia; 12 Dengue Branch, Centers for Disease Control and Prevention, San Juan, Puerto Rico, United States of America; 13 Bouvé College of Health Sciences and Network Science Institute, Northeastern University, Boston, Massachusetts, United States of America; 14 Mahidol Oxford Tropical Medicine Research Unit, Mahidol University, Bangkok, Thailand; 15 Centre for Tropical Medicine and Global Health, Nuffield Department of Medicine, University of Oxford, Oxford, United Kingdom; 16 The Open University, Milton Keynes, United Kingdom; 17 School of Public Health, University of Hong Kong, Hong Kong, Hong Kong; 18 School of Geography and the Environment, University of Oxford, Oxford, United Kingdom; 19 Biology Department, Stanford University, Stanford, California, United States of America; 20 Biodiversity Institute, The University of Kansas Biodiversity Institute and Natural History Museum, Lawrence, Kansas, United States of America; 21 Department of Medicine, University of California San Francisco, San Francisco, California, United States of America; 22 Department of Epidemic and Pandemic Preparedness and Prevention, World Health Organization, Geneva, Switzerland; 23 Department of Geography and the Emerging Pathogens Institute, University of Florida, Gainesville, Florida, United States of America; 24 Department of Genetics, University of Cambridge, Cambridge, United Kingdom; 25 Heidelberg Institute of Global Health, University of Heidelberg: Universitat Heidelberg, Heidelberg, Germany; 26 Department of Public Health and Clinical Medicine, Umeå University, Umeå, Sweden; CNRS, FRANCE

## Abstract

Global risk maps are an important tool for assessing the global threat of mosquito and tick-transmitted arboviral diseases. Public health officials increasingly rely on risk maps to understand the drivers of transmission, forecast spread, identify gaps in surveillance, estimate disease burden, and target and evaluate the impact of interventions. Here, we describe how current approaches to mapping arboviral diseases have become unnecessarily siloed, ignoring the strengths and weaknesses of different data types and methods. This places limits on data and model output comparability, uncertainty estimation and generalisation that limit the answers they can provide to some of the most pressing questions in arbovirus control. We argue for a new generation of risk mapping models that jointly infer risk from multiple data types. We outline how this can be achieved conceptually and show how this new framework creates opportunities to better integrate epidemiological understanding and uncertainty quantification. We advocate for more co-development of risk maps among modellers and end-users to better enable risk maps to inform public health decisions. Prospective validation of risk maps for specific applications can inform further targeted data collection and subsequent model refinement in an iterative manner. If the expanding use of arbovirus risk maps for control is to continue, methods must develop and adapt to changing questions, interventions and data availability.

## Introduction

Global and regional risk maps of disease play an increasingly prominent role in health policy and decision making. For arboviruses, risk maps have become integral to estimating global distribution and burden [1,2], future projections [3,4], outbreak early warning systems [5,6], targeting of interventions [2,7], assessments of intervention cost effectiveness [8] and evaluation of the progress towards international goals. These needs have become increasingly acute as the global burden of dengue has ballooned to an estimated 57–390 million infections per year [1,2,9] with increasingly large outbreaks fuelled by urbanisation, travel and climate change [10]. Other *Aedes* mosquito-borne viruses (Zika, chikungunya and yellow fever), *Culex* mosquito-borne viruses (Japanese encephalitis, Rift Valley fever, West Nile fever) and tick-borne viruses (Tick-borne encephalitis, Crimean-Congo Haemorrhagic Fever) share many of the same environmental drivers and recent increases in incidence and range [11,12].

The World Health Organization Global Arbovirus Initiative (WHO GAI) [13] has identified risk mapping as a key evidence gap within the surveillance pillar, and a recent project, conducted by many of the same authors as this manuscript, has aimed to fill this gap. First, a systematic review [14] characterised the current state of the art for arbovirus risk mapping and gave key recommendations to improve current practice. Second, new global risk maps were generated for dengue, chikungunya, Zika and yellow fever that added 42,134 new contemporary data points and standardised methodology to improve comparability among arbovirus risk maps and address surveillance biases [15]. In this perspective manuscript, we aim to look further ahead and identify the key methodological leaps that need to be made to realise the range of new applications of risk maps for arbovirus control. While we focus primarily on global mapping of *Aedes*-borne arboviruses, the identified gaps and proposed solutions are also applicable to other viral diseases spread by arthropods.

## Different types of data measure different types of risk

**Fig 1:**
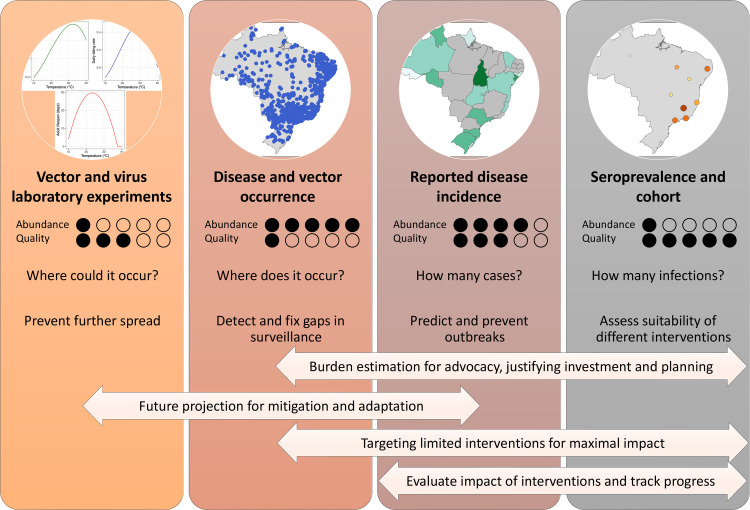
The main types of data currently used for arbovirus disease mapping. For each data type, the figure summarises relative abundance and quality of data (bias, representativeness and consistency among areas and over time) (as assessed by author consensus, both rated 1–5), the main questions each data type aims to answer and associated public health actions that can be undertaken with such knowledge. Use cases that can draw on multiple categories are labelled in bi-directional arrows at the bottom of the figure.

Models based on different types of data inform different types of risk which have different, but often overlapping, applications (Fig 1). The most common types of risk predicted in arbovirus risk maps are: risk of establishing local transmission if introduced, risk of ongoing transmission, risk of disease and risk of infection, each with their own area or population denominator and unit of time (Fig 1) [14]. To estimate risk in areas where arbovirus data are not available or are inadequate, risk mapping models integrate data on climate, built and natural environments, human mobility, interventions, and sociodemographics to identify correlates of arbovirus risk that are then used to make predictions and projections.

Data from laboratory experiments on mosquitoes can be used to understand how climate variables, notably temperature, affect critical steps in arbovirus transmission [16]. Controlled laboratory conditions enable precise measurements of these effects, but experiments are performed on minority of mosquito lineages [17] and are unrepresentative of field conditions [4,18]. These physiological studies can inform which areas may be vulnerable to outbreaks [19], and predict how future climate change may influence the global limits of transmission [4,18,20].

Occurrence data [21] (presence/absence of disease in a precise location) are the most abundant form of data, and often are the only measure of risk available in data-sparse regions, making occurrence data an appropriate choice for mapping the current global distributions of arboviral diseases. Occurrence data, however, tells us little about the magnitude of risk of transmission and is affected by spatial differences in surveillance intensity. Occurrence data for vectors or reservoir species can also inform maps of disease invasion, emergence and transmission risk [22].

Case incidence data is available from many arbovirus-endemic countries and measures changing risk over time, but can be difficult to compare among countries due to differing case detection, diagnosis and reporting criteria [23]. Despite these limitations, maps based on reported incidence data give the best estimates of clinical burden [24,25] and treatment resource need, and provide the most time-sensitive and up-to-date estimates of risk, enabling the development of outbreak forecasting systems [5,6] and the evaluation of intervention impact [26]. Vector abundance data (mosquito counts over time) can also inform time-varying estimates of risk, especially in non-endemic settings with lower case counts.

Age-stratified seroprevalence data [27,28] and serological cohort studies [1], can be analysed to reconstruct a long-term history of past infection dynamics, but have coarse temporal resolution and are geographically sparse and rare in low-transmission areas. As the only types of data that measure arbovirus infections (as opposed to disease), they are the only type of data able to inform the full extent of disease burden and transmission intensity. Estimates of long-term average force of infection can also be obtained by analysing the age distribution of cases and/or severe cases provided appropriate validation with seroprevalence data [29,30].

Different approaches intentionally aim to predict different elements of transmission risk that, depending on the setting, may not correlate with one another. In many cases, choice of approach may depend on the availability of information or end-user needs. Indeed, answers to some of the biggest questions on global burden, future change and intervention impact can come from multiple separate approaches (Fig 1), at times with contrasting results that can be challenging to interpret.

## Main limitations of current mapping approaches and why they matter

Because each type of data, in isolation, has sizeable gaps in space and/or time many mapping approaches aggregate data over longer time periods to give a snapshot estimate of long-term average risk. This limits a model’s ability to map detailed changes in risk over time and means these approaches often cannot directly attribute such changes to variation in climate, urbanisation, travel, and intervention use, although some national and regional examples are beginning to emerge [31]. With the rapid development of new vaccines and vector control tools and a growing demand for climate change attribution studies, novel temporally dynamic maps with robust mechanistic links to causal drivers are increasingly needed.

### Data comparability

Even when data are available over long time periods and broad geographic regions, they may not be comparable due to variable surveillance, diagnosis, and reporting practices, an issue that is particularly acute for arboviruses due to non-specific disease symptoms [32]. Without appropriate adjustment, this may result in risk maps representing surveillance sensitivity and specificity rather than, as intended, transmission risk. Key vulnerabilities include comparisons of incidence data among countries with different surveillance systems and seroprevalence data measured with different assays (especially in areas with Zika, or other related viruses, due to cross reactive antibodies). Global risk maps will only offer predictive advantages over local risk maps if issues with data comparability can be addressed in the modelling approach. Occurrence data models can be designed to account for the inherent spatial biases in occurrence data through different methods of background or pseudo-absence point generation, but too often random selection is used [14] (which assumes uniform surveillance) despite increasingly advanced methods that nest surveillance models in a hierarchical structure being available [29].

### Prediction uncertainty

Current modelling approaches also often systematically underrepresent uncertainty, leading to overly precise risk estimates and overconfident comparisons of risk among areas. Existing frameworks are rarely capable of quantifying spatial uncertainty due to geographic gaps in data, leading to highly confident predictions in areas such as Africa despite sparse data coverage [33–35]. Choice of co-variables, their data sources, their methods of processing and extrapolation in addition to their validated accuracy are all potential areas of uncertainty that are rarely propagated. A better assessment of uncertainty informs data collection and iterative model improvement, but is also critical if such maps are to be used to make decisions on, for example, intervention targeting. Visualising uncertainty in an interpretable way on maps is challenging and current portrayals can lead to overconfident uses of the maps and, on occasion, misinterpretation of the raw data.

### Generalisability

Machine learning methods have increased the complexity of the relationships we are able to fit between variables and disease risk and often appear to give superior predictive performance when evaluated via internal cross validation [14]. One consequence of this flexibility, however, is less opportunity to include prior biological, ecological and epidemiological understanding of the processes that shape the spatial distributions of these viruses. This can lead to poor generalisability with far inferior performance when evaluated against out of sample data. This is particularly acute in data sparse areas or in areas with environmental characteristics far outside the observed range, which happen to be where the largest changes in future risk are projected, for example, dengue in Africa [36]. Despite this, few models are evaluated with prospective data collection [37].

## Priority areas for model development

The next generation of arbovirus risk mapping models must formally integrate different types of data to overcome the limitations discussed above and, thus, realise this new range of applications. As a collateral benefit, joint inference approaches also open opportunities for more integration of ecological and epidemiological understanding that aim to better represent the mechanisms that link risk factors to transmission. To achieve these the following priority areas will need to be addressed:

### Leveraging strengths of different data types in a combined modelling framework

New models need to be formulated with a joint likelihood where transmission intensity is inferred at a high spatial and temporal resolution as a function of combined occurrence, incidence and seroprevalence data. Such an approach would take advantage of the more accurate long-term estimates of force of infection from seroprevalence data, but also characterise the high spatial and temporal heterogeneity measured through occurrence and incidence data, respectively. Currently, few areas exist globally where these three data sources overlap at scales and resolutions that would make this kind of joint estimation possible. Geospatial models will, therefore, be important for spatially projecting estimates of risk from each type of data across common areas. This geospatial model would need to be nested within the joint inference model through a hierarchical structure. Appropriately estimating and weighting uncertainty from the original data, the modifiable areal unit problem, extrapolation of the data and assessing the degree of consensus among different types of data will be key challenges, but examples from malaria risk mapping where case incidence and prevalence data are increasingly combined show that they can be overcome [38,39]. This new modelling approach will likely not work everywhere, but discovering that data from a particular area are too sparse to characterise transmission is, in itself, an informative result.

A useful intermediate step would be a systematic comparison of risk maps derived from different approaches across different geographic and transmission strata. This analysis could assess consensus and better understand which types of risk are captured by different types of data in different settings [40]. These hypotheses can be tested in a data-driven manner through model “stacking” wherein predictions from one model are tested for inclusion as a relevant covariate in another model [41].

Longer-term, joint inference models could incorporate the growing volume of arbovirus genomic data to improve estimates of risk during emergence [42] or stratify risk by different viral lineages and the fields of phylodynamics and phylogenetics offer examples of joint models that could guide development of the models proposed here [43]. Joint inference models could also be used to combine data sets across arboviruses, leveraging their shared mosquito vectors and similar spatial and seasonal drivers, but different levels of immunity and distinct emergence histories [44]. Achieving this integration will require directly accounting for additional measurement error due to misdiagnosis and antibody cross reaction. This step would not only improve estimates for the major arboviral diseases by drawing on more data, but could also be leveraged to predict the potential spread of new emerging arboviruses with pandemic potential.

### Incorporating biological, ecological and epidemiological understanding with the use of (semi) mechanistic risk functions and constraints

The next generation of arbovirus risk models should include more biologically meaningful constraints on the relationships between covariates and risk. The Ross-MacDonald equation for the reproduction number (*R*) of a vector borne disease can be used to explore such opportunities:


$$R=S\frac{ma^{2}bcp^{n}}{r\left(-\ln\left(p\right)\right)}$$

Studying the effects of temperature mosquitoes in the laboratory has allowed parameterisation of functional relationships between temperature and daily mosquito survival rate (*p*), virus incubation period in the mosquito (*n*), human biting rate (*a*) and mosquito–host transmission rates (b,c) [45–47]. Humidity is also a key driver of mosquito survival (and possibly biting rate) and can be incorporated in a similar way [48]. With growing availability of species distribution models for medically important vector species, there may be new opportunities to estimate vector abundance (and thus mosquitoes per person *m*). Rather than using the direct estimates of occurrence probabilities that come from such models, some studies have suggested that measures of niche centrality (i.e., how closely an environments’ characteristics are to the “ideal” environment) calibrated to field estimates may more accurately estimate abundance [49].

The Ross-Macdonald equation also provides a framework for understanding how different vector species (or sub-species) jointly contribute to transmission. Incorporating covariates that summarise the different epidemiologically relevant bionomics of *Aedes aegypti* and *Aedes albopictus* may improve risk maps in emerging settings, such as Europe where only *Ae. albopictus* is present, and explain how risk changes when species-specific interventions, such as *wMel Wolbachia*, are used at scale. Genetic or phenotypic differences in key mosquito bionomics (particularly b,c,a and *n*), may explain broad regional differences in transmission risk, such as comparatively low reported incidence of dengue and Zika in Africa compared with Asia and the Americas [50].

**Fig 2:**
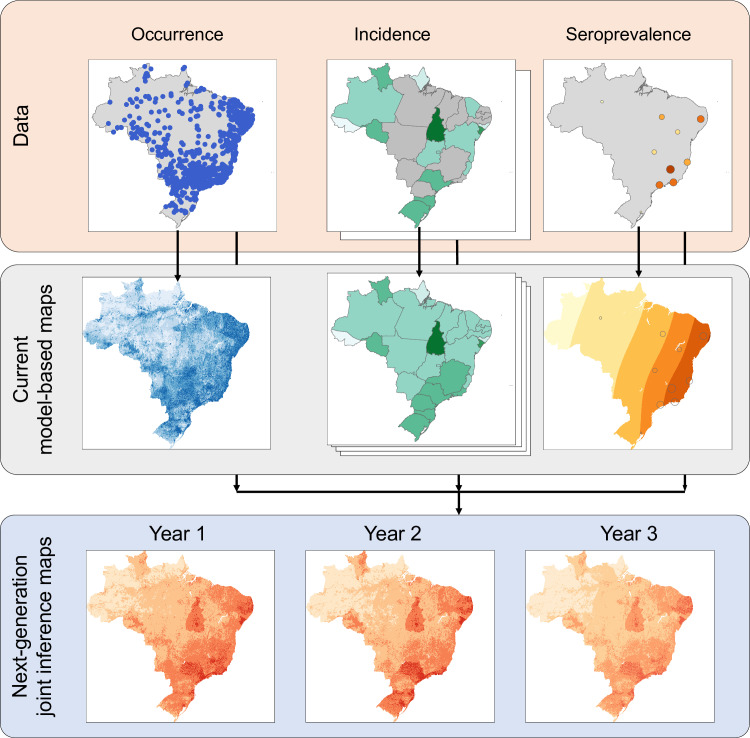
Conceptual overview of a joint inference mapping approach showing example occurrence, incidence and seroprevalence data for Brazil (top row), the kinds of risk maps that can be generated from each of these data sets independently using current generation methods (middle row) and the time-varying more accurate maps that could be generated from a joint-inference modelling approach (bottom row, uses a simple equal weight ensemble for illustration purposes only).

While most current mapping models focus exclusively on environmental drivers of transmission risk, human susceptibility *S* to infection and, to a lesser extent, duration of human infectiousness *r* play an increasingly important role. Dengue virus serotype, genotype and infection sequence all affect disease severity while current vaccines are inconsistently implemented and show variable effectiveness by serotype [51,52]. Incorporating this dynamic landscape of susceptibility must become a priority if models are to inform vaccination strategy and burden estimation. Some advances in this area have been made in yellow fever where demographic models and vaccination data have been integral to mapping risk [53,54]. Combining such dynamic models with a joint inference framework (Fig 2) can better inform the relationship between types of risk, disease incidence and seroprevalence. The next generation for arbovirus burden estimation and future projection models should include the mitigating effects of accumulating immunity, including how shifting future birth, death and migration rates affect population-wide immunity and susceptibility to severe disease outcomes.

### Improving methods to measure, propagate and visualise uncertainty and validation of map predictions

Appropriate measures of uncertainty are essential for maps to inform public health decisions and, increasingly, target further data collection. The highest priority is to more accurately convey uncertainty related to geographic gaps in epidemiological data, particularly in Africa. This most commonly occurs because models rarely account for spatial autocorrelation in the data in their model formulation, instead relying on environmental variables alone. Accounting for spatial autocorrelation in the data, using random effects structed by proximity or mobility, provides a more robust and statistically valid test for models and are already commonplace in analyses of incidence data [55,56]. Sharing the full distribution of uncertainty in risk maps for every geographic area can empower local modellers by providing priors for variables that are not collected or difficult to estimate. Disaggregating uncertainty into its constituent parts (data, covariates, model structure, projection) will also help prioritise model refinement efforts.

When arbovirus transmission risk can be robustly theoretically and empirically linked to climatic, environmental, and socioeconomic drivers, it is important that uncertainty in estimation and extrapolation of these, often model-estimated, drivers is propagated. This is particularly acute with future projection studies where multiple climate models project multiple scenarios. More coordinated efforts are required to align projections of climate with those of demographics, urbanisation and migration [35]. For now, generating separate arbovirus risk projections for each scenario and climate model combination is a comprehensive, if computationally expensive, solution. Sharing data, predictions, and code via online repositories and platforms (e.g. Figshare, [30], Github, https://github.com) enables downstream analyses to better propagate scenario uncertainty (e.g. https://dataverse.harvard.edu/dataverse/Aedesmaps).

Even the formulation of the risk mapping model itself introduces a range of uncertain assumptions. Choice of covariates (or covariate selection procedures) and the degree of flexibility in the relationship between covariates and disease risk introduce assumptions that could be better routinely clarified using conceptual frameworks, guided by the literature and/or directed acyclic graphs. Ensembles of multiple plausible mapping model structures would bring the field in line with developments in temporal forecasting [5,57].

Communicating prediction uncertainty through maps remains an open challenge in the field of spatial epidemiology. While some innovative visualisation approaches have been proposed including pixelating more uncertain areas [58] and use of two dimensional bi-variate choropleth maps [59], the best solution will depend on the specific use case of the map. Summarising probabilistic model predictions around a policy-relevant threshold (e.g. 80% certain that prevalence is above 70%) has proven an effective approach for informing mass drug administration decisions for other neglected tropical diseases [60].

Finally, validation of risk maps needs to become an iterative, multidisciplinary exercise that involves out-of-sample validation including prospective data collection. Out-of-sample validation can be accomplished by collecting or accessing new data, and gives a more accurate measure of model utility for areas without data, i.e. the areas where risk estimates are most valuable. Validation is not solely an exercise to prove the map right or wrong, but an opportunity to discuss with stakeholders across multiple levels of government the strength of evidence for arbovirus risk in different areas, strengthen surveillance and to identify key data or model gaps that could improve estimates. Interactive dashboards can facilitate this. Currently, arbovirus risk maps are validated through comparison with different measures of risk (Fig 1) [61,62] or periodically as more of the same types of data are published [63,64]. We are not aware of any published examples where a clear study design has been used for the purpose of prospective data collection to validate a disease risk map, but examples from species distribution mapping in ecology provide a framework that could be adapted for disease risk maps [65]. Further research is needed into disease-specific approaches to risk map validation. An arbovirus risk mapping project planned today should consider how structured involvement of local experts with a diverse range of expertise and potential map use cases could be used to evaluate the reliability and usefulness of risk maps.

## Key data needs

The full benefits of these new models will only be realised if they are accompanied by investments in data collection, standardisation, and accessibility. High-resolution global maps should not give the illusion that further data collection is not valuable. Models can generate maps that target data collection in specific sites to fill geographic and/or environmental gaps [39]. Investment in databases to collate, standardise and make accessible arbovirus data are also important with initiatives including OpenDengue [23] (https://opendengue.org), ArboTracker [66] (https://new.serotracker.com/pathogen/arbovirus/dashboard) and the WHO Global dengue surveillance dashboard (https://worldhealthorg.shinyapps.io/dengue_global/) making progress. Similar resources for roll out of new vaccines and *Wolbachia* replacement technologies will need to be developed and lessons can be learned from yellow fever, where databases of vaccine coverage have been pivotal for understanding the distribution of the disease [67,68]. New covariates for human mobility, water and waste management infrastructure and other dimensions of the built environment at the global scale would improve estimates of risk [10]. Increasing the temporal resolution of covariates for current and historical periods is also urgently needed.

## Conclusions

As arboviral diseases have emerged and re-emerged as global public health threats, a growing range of approaches to map arbovirus risk have proliferated. While these maps have been useful, we are approaching the limits of what can be achieved with current methods. Here, we call for the development of a new generation of modelling frameworks that integrate the strengths of different types of data, incorporate more epidemiological understanding and appropriately quantify uncertainty. These new joint inference mapping models could enable a significant advance in how risk maps are used by arbovirus control programmes at a time when they need to make difficult decisions about investment in novel vector control tools and vaccines.

## References

[1] Bhatt S, Gething PW, Brady OJ, Messina JP, Farlow AW, Moyes CL, et al. The global distribution and burden of dengue. Nature. 2013;496(7446):504–7. doi: 10.1038/nature12060 23563266 PMC3651993

[2] Cattarino L, Rodriguez-Barraquer I, Imai N, Cummings DAT, Ferguson NM. Mapping global variation in dengue transmission intensity. Sci Transl Med. 2020;12(528):eaax4144. doi: 10.1126/scitranslmed.aax4144 31996463

[3] Messina JP, Brady OJ, Golding N, Kraemer MUG, Wint GRW, Ray SE, et al. The current and future global distribution and population at risk of dengue. Nat Microbiol. 2019;4(9):1508–15. doi: 10.1038/s41564-019-0476-8 31182801 PMC6784886

[4] Ryan SJ, Carlson CJ, Mordecai EA, Johnson LR. Global expansion and redistribution of Aedes-borne virus transmission risk with climate change. PLoS Negl Trop Dis. 2019;13(3):e0007213. doi: 10.1371/journal.pntd.0007213 30921321 PMC6438455

[5] Colón-González FJ, Soares Bastos L, Hofmann B, Hopkin A, Harpham Q, Crocker T, et al. Probabilistic seasonal dengue forecasting in Vietnam: a modelling study using superensembles. Cook AR, editor. PLoS Med. 2021;18(3):e1003542. doi: 10.1371/journal.pmed.1003542 33661904 PMC7971894

[6] Lauer SA, Sakrejda K, Ray EL, Keegan LT, Bi Q, Suangtho P, et al. Prospective forecasts of annual dengue hemorrhagic fever incidence in Thailand, 2010-2014. Proc Natl Acad Sci U S A. 2018;115(10):E2175–82. doi: 10.1073/pnas.1714457115 29463757 PMC5877997

[7] O’Reilly KM, Hendrickx E, Kharisma DD, Wilastonegoro NN, Carrington LB, Elyazar IRF, et al. Estimating the burden of dengue and the impact of release of wMel Wolbachia-infected mosquitoes in Indonesia: a modelling study. BMC Med. 2019;17(1):172. doi: 10.1186/s12916-019-1396-4 31495336 PMC6732838

[8] Brady OJ, Kharisma DD, Wilastonegoro NN, O’Reilly KM, Hendrickx E, Bastos LS, et al. The cost-effectiveness of controlling dengue in Indonesia using wMel Wolbachia released at scale: a modelling study. BMC Med. 2020;18(1):186. doi: 10.1186/s12916-020-01638-2 32641039 PMC7346418

[9] Vos T, Lim SS, Abbafati C, Abbas KM, Abbasi M, Abbasifard M, et al. Global burden of 369 diseases and injuries in 204 countries and territories, 1990–2019: a systematic analysis for the Global Burden of Disease Study 2019. Lancet. 2020;396:1204–22. doi: 10.1016/S0140-6736(20)30925-9 33069326 PMC7567026

[10] Gibb R, Colón-González FJ, Lan PT, Huong PT, Nam VS, Duoc VT, et al. Interactions between climate change, urban infrastructure and mobility are driving dengue emergence in Vietnam. Nat Commun. 2023;14(1):8179. doi: 10.1038/s41467-023-43954-0 38081831 PMC10713571

[11] Gilbert L. The Impacts of Climate Change on Ticks and Tick-Borne Disease Risk. Annu Rev Entomol. 2021;66:373–88. doi: 10.1146/annurev-ento-052720-094533 33417823

[12] Farooq Z, Rocklöv J, Wallin J, Abiri N, Sewe MO, Sjödin H, et al. Artificial intelligence to predict West Nile virus outbreaks with eco-climatic drivers. Lancet Reg Health Eur. 2022;17:100370. doi: 10.1016/j.lanepe.2022.100370 35373173 PMC8971633

[13] Balakrishnan VS. WHO launches global initiative for arboviral diseases. Lancet Microbe. 2022;3(6):e407–e407. doi: 10.1016/S2666-5247(22)00130-6 35659901 PMC9159734

[14] Lim A-Y, Jafari Y, Caldwell JM, Clapham HE, Gaythorpe KAM, Hussain-Alkhateeb L, et al. A systematic review of the data, methods and environmental covariates used to map Aedes-borne arbovirus transmission risk. BMC Infect Dis. 2023;23(1):708. doi: 10.1186/s12879-023-08717-8 37864153 PMC10588093

[15] Lim A, Shearer F, Sewalk K, Pigott D, Clarke J, Ghouse A, et al. The overlapping global distribution of dengue, chikungunya, Zika and yellow fever. 2024. 10.1038/s41467-025-58609-5 PMC11986131 40210848

[16] Mordecai EA, Caldwell JM, Grossman MK, Lippi CA, Johnson LR, Neira M, et al. Thermal biology of mosquito‐borne disease. Ecol Lett. 2019;22(10):1690–708. doi: 10.1111/ele.13335 31286630 PMC6744319

[17] Chen B, Sweeny AR, Wu VY, Christofferson RC, Ebel G, Fagre AC, et al. Exploring the Mosquito–Arbovirus network: a survey of vector competence experiments. Am J Trop Med Hyg. 2023;108(5):987–94. doi: 10.4269/ajtmh.22-0511 37037424 PMC10160896

[18] Ryan SJ, Carlson CJ, Tesla B, Bonds MH, Ngonghala CN, Mordecai EA, et al. Warming temperatures could expose more than 1.3 billion new people to Zika virus risk by 2050. Global Change Biol. 2021;27(1):84–93. doi: 10.1111/gcb.15384 33037740 PMC7756632

[19] Andronico A, Menudier L, Salje H, Vincent M, Paireau J, de Valk H, et al. Comparing the performance of three models incorporating weather data to forecast dengue epidemics in Reunion Island, 2018–2019. J Infect Dis. 2024;229(1):10–8. doi: 10.1093/infdis/jiad468 37988167 PMC10786251

[20] Ryan SJ. Mapping thermal physiology of vector-borne diseases in a changing climate: shifts in geographic and demographic risk of suitability. Curr Environ Health Rep. 2020;7(4):415–23. doi: 10.1007/s40572-020-00290-5 32902817 PMC7748992

[21] Messina JP, Brady OJ, Pigott DM, Brownstein JS, Hoen AG, Hay SI. A global compendium of human dengue virus occurrence. Sci Data. 2014;1:140004. doi: 10.1038/sdata.2014.4 25977762 PMC4322574

[22] Kraemer MUG, Reiner RC, Brady OJ, Messina JM, Bisanzio D, Perkins TA, et al. Modelling the past and future spread of the arbovirus vectors *Aedes aegypti* and *Aedes albopictus*. Nat Microbiol. 2019;4:854–63. 30833735 10.1038/s41564-019-0376-y PMC6522366

[23] Clarke J, Lim A, Gupte P, Pigott DM, Van Panhuis WG, Brady OJ. A global dataset of publicly available dengue case count data. Sci Data. 2024;11(1):296. doi: 10.1038/s41597-024-03120-7 38485954 PMC10940302

[24] Stanaway JD, Shepard DS, Undurraga EA, Halasa YA, Coffeng LE, Brady OJ, et al. The global burden of dengue: an analysis from the Global Burden of Disease Study 2013. Lancet Infect Dis. 2016;16(6):712–23. doi: 10.1016/S1473-3099(16)00026-8 26874619 PMC5012511

[25] World Health Organization. A Toolkit for national dengue burden estimation. Geneva; 2018. Available from: https://www.who.int/publications/i/item/WHO-CDS-NTD-VEM-2018.05

[26] Ribeiro Dos Santos G, Durovni B, Saraceni V, Souza Riback TI, Pinto SB, Anders KL, et al. Estimating the effect of the wMel release programme on the incidence of dengue and chikungunya in Rio de Janeiro, Brazil: a spatiotemporal modelling study. Lancet Infect Dis. 2022;22:1587–95. doi: 10.1016/S1473-3099(22)00436-4 36182679 PMC9630156

[27] Imai N, Dorigatti I, Cauchemez S, Ferguson NM. Estimating dengue transmission intensity from sero-prevalence surveys in multiple countries. PLoS NeglTrop Dis. 2015;9(4):e0003719. doi: 10.1371/journal.pntd.0003719 25881272 PMC4400108

[28] Kang H, Auzenbergs M, Clapham H, Maure C, Kim J-H, Salje H, et al. Chikungunya seroprevalence, force of infection, and prevalence of chronic disability after infection in endemic and epidemic settings: a systematic review, meta-analysis, and modelling study. Lancet Infect Dis. 2024;24(5):488–503. doi: 10.1016/S1473-3099(23)00810-1 38342105

[29] Rodriguez-Barraquer I, Salje H, Cummings DA. Opportunities for improved surveillance and control of dengue from age-specific case data. eLife. 2019;8:e45474. doi: 10.7554/eLife.45474 31120419 PMC6579519

[30] Nemoto T, Aubry M, Teissier Y, Paul R, Cao-Lormeau V-M, Salje H, et al. Reconstructing long-term dengue virus immunity in French Polynesia. PLoS Negl Trop Dis. 2022;16(10):e0010367. doi: 10.1371/journal.pntd.0010367 36191046 PMC9560594

[31] Childs ML, Lyberger K, Harris M, Burke M, Mordecai EA. Climate warming is expanding dengue burden in the Americas and Asia. 2024. 10.1073/pnas.2512350122 PMC12452855 40924448

[32] Sarti E, L’Azou M, Mercado M, Kuri P, Siqueira JB, Solis E, et al. A comparative study on active and passive epidemiological surveillance for dengue in five countries of Latin America. Int J Infect Dis. 2016;44:44–9. doi: 10.1016/j.ijid.2016.01.015 26836763

[33] Ryan SJ, Lippi CA, Caplan T, Diaz A, Dunbar W, Grover S, et al. The current landscape of software tools for the climate-sensitive infectious disease modelling community. Lancet Planet Health. 2023;7(6):e527–36. doi: 10.1016/S2542-5196(23)00056-6 37286249

[34] Musa SM, Haruna UA, Manirambona E, Eshun G, Ahmad DM, Dada DA, et al. Paucity of health data in Africa: an obstacle to digital health implementation and evidence-based practice. Public Health Rev. 2023;44:1605821. doi: 10.3389/phrs.2023.1605821 37705873 PMC10495562

[35] Reidpath DD, Allotey P. The problem of ‘trickle-down science’ from the Global North to the Global South. BMJ Glob Health. 2019;4(4):e001719. doi: 10.1136/bmjgh-2019-001719 31406597 PMC6666820

[36] Messina JP, Brady OJ, Golding N, Kraemer MUG, Wint GRW, Ray SE, et al. The current and future global distribution and population at risk of dengue. Nat Microbiol. 2019;4:1508–15. 31182801 10.1038/s41564-019-0476-8 PMC6784886

[37] Lim A-Y, Jafari Y, Caldwell JM, Clapham HE, Gaythorpe KAM, Hussain-Alkhateeb L, et al. A systematic review of the data, methods and environmental covariates used to map Aedes-borne arbovirus transmission risk. BMC Infect Dis. 2023;23(1):708–708. doi: 10.1186/s12879-023-08717-8 37864153 PMC10588093

[38] Arambepola R, Keddie SH, Collins EL, Twohig KA, Amratia P, Bertozzi-Villa A, et al. Spatiotemporal mapping of malaria prevalence in Madagascar using routine surveillance and health survey data. Sci Rep. 2020;10(1):18129. doi: 10.1038/s41598-020-75189-0 33093622 PMC7581764

[39] Arambepola R, Lucas TCD, Nandi AK, Gething PW, Cameron E. A simulation study of disaggregation regression for spatial disease mapping. Stat Med. 2022;41(1):1–16. doi: 10.1002/sim.9220 34658042

[40] Monaghan AJ, Eisen RJ, Eisen L, McAllister J, Savage HM, Mutebi J-P, et al. Consensus and uncertainty in the geographic range of *Aedes aegypti* and *Aedes albopictus* in the contiguous United States: multi-model assessment and synthesis. PLoS Comput Biol. 2019;15(10):e1007369. doi: 10.1371/journal.pcbi.1007369 31600194 PMC6786520

[41] Bhatt S, Cameron E, Flaxman SR, Weiss DJ, Smith DL, Gething PW. Improved prediction accuracy for disease risk mapping using Gaussian process stacked generalization. J R Soc Interface. 2017;14(134):20170520. doi: 10.1098/rsif.2017.0520 28931634 PMC5636278

[42] Hill SC, Dellicour S, Claro IM, Sequeira PC, Adelino T, Thézé J, et al. Climate and land-use shape the spread of zoonotic yellow fever virus. medRxiv. 2022;2022.08.25.22278983. doi: 10.1101/2022.08.25.22278983

[43] Judge C, Vaughan T, Russell T, Abbott S, Plessis L du, Stadler T, et al. EpiFusion: Joint inference of the effective reproduction number by integrating phylodynamic and epidemiological modelling with particle filtering. PLoS Comput Biol. 2024;20:e1012528. doi: 10.1371/journal.pcbi.1012528 39527637 PMC11581393

[44] Shearer FM, Huang Z, Weiss DJ, Wiebe A, Gibson HS, Battle KE, et al. Estimating geographical variation in the risk of zoonotic plasmodium knowlesi infection in countries eliminating malaria. PLoS Negl Trop Dis. 2016;10(8):e0004915. doi: 10.1371/journal.pntd.0004915 27494405 PMC4975412

[45] Tesla B, Demakovsky LR, Mordecai EA, Ryan SJ, Bonds MH, Ngonghala CN, et al. Temperature drives Zika virus transmission: evidence from empirical and mathematical models. Proc Biol Sci. 2018;285(1884):20180795. doi: 10.1098/rspb.2018.0795 30111605 PMC6111177

[46] Mordecai EA, Cohen JM, Evans MV, Gudapati P, Johnson LR, Lippi CA, et al. Detecting the impact of temperature on transmission of Zika, dengue, and chikungunya using mechanistic models. PLoS Negl Trop Dis. 2017;11(4):e0005568. doi: 10.1371/journal.pntd.0005568 28448507 PMC5423694

[47] Brady OJ, Johansson MA, Guerra CA, Bhatt S, Golding N, Pigott DM, et al. Modelling adult *Aedes aegypti* and *Aedes albopictus* survival at different temperatures in laboratory and field settings. Parasit Vectors. 2013;6:351–351. doi: 10.1186/1756-3305-6-351 24330720 PMC3867219

[48] Brown JJ, Pascual M, Wimberly MC, Johnson LR, Murdock CC. Humidity – The overlooked variable in the thermal biology of mosquito-borne disease. Ecol Lett. 2023;26(7):1029–49. doi: 10.1111/ele.14228 37349261 PMC10299817

[49] Osorio‐Olvera L, Yañez‐Arenas C, Martínez‐Meyer E, Peterson AT. Relationships between population densities and niche‐centroid distances in North American birds. Enquist B, editor. Ecol Lett. 2020;23(3):555–64. doi: 10.1111/ele.13453 31944513

[50] Caldwell J, Lambrechts L, Rose NH. Vector population variation shapes the present and future of Zika virus transmission patterns in Africa. Rochester, NY; 2023. 10.1016/S2542-5196(24)00276-6 PMC12352338 39674192

[51] Katzelnick LC, Gresh L, Halloran ME, Mercado JC, Kuan G, Gordon A, et al. Antibody-dependent enhancement of severe dengue disease in humans. Science (New York, NY). 2017;358:929–32. 10.1126/science.aan6836 PMC5858873 29097492

[52] Biswal S, Borja-Tabora C, Martinez Vargas L, Velásquez H, Theresa Alera M, Sierra V, et al; TIDES study group. Efficacy of a tetravalent dengue vaccine in healthy children aged 4-16 years: a randomised, placebo-controlled, phase 3 trial. Lancet. 2020;395(10234):1423–33. doi: 10.1016/S0140-6736(20)30414-1 32197105

[53] Shearer FM, Moyes CL, Pigott DM, Brady OJ, Marinho F, Deshpande A, et al. Global yellow fever vaccination coverage from 1970 to 2016: an adjusted retrospective analysis. Lancet Infect Dis. 2017;17(11):1209–17. doi: 10.1016/S1473-3099(17)30419-X 28822780 PMC5666204

[54] Childs ML, Nova N, Colvin J, Mordecai EA. Mosquito and primate ecology predict human risk of yellow fever virus spillover in Brazil. Philos Trans R Soc Lond Ser B. 2019;374(1782):20180335. doi: 10.1098/rstb.2018.0335 31401964 PMC6711306

[55] Gibb R, Colón-González FJ, Lan PT, Huong PT, Nam VS, Duoc VT, et al. Interactions between climate change, urban infrastructure and mobility are driving dengue emergence in Vietnam. Nat Commun. 2023;14(1):8179–8179. doi: 10.1038/s41467-023-43954-0 38081831 PMC10713571

[56] Benito BM. spatialRF: easy spatial regression with random forest (1.1.0). Zenodo; 2021.

[57] Reich NG, Lessler J, Funk S, Viboud C, Vespignani A, Tibshirani RJ, et al. Collaborative hubs: making the most of predictive epidemic modeling. Am J Public Health. 2022;112(6):839–42. doi: 10.2105/AJPH.2022.306831 35420897 PMC9137029

[58] Taylor AR, Watson JA, Buckee CO. Pixelate to communicate: visualising uncertainty in maps of disease risk and other spatial continua. arXiv. 2020; doi: 10.48550/arXiv.2005.11993

[59] Golding N, Burstein R, Longbottom J, Browne AJ, Fullman N, Osgood-Zimmerman A, et al. Mapping under-5 and neonatal mortality in Africa, 2000–15: a baseline analysis for the sustainable development goals. Lancet. 2017;390(10108):2171–82. doi: 10.1016/s0140-6736(17)31758-0 28958464 PMC5687451

[60] Fronterre C, Amoah B, Giorgi E, Stanton MC, Diggle PJ. Design and analysis of elimination surveys for neglected tropical diseases. J Infect Dis. 2020;221(Suppl 5):S554–60. doi: 10.1093/infdis/jiz554 31930383 PMC7289555

[61] Balmori-de la Puente A, Rodríguez-Escolar I, Collado-Cuadrado M, Infante González-Mohino E, Vieira Lista MC, Hernández-Lambraño RE, et al. Transmission risk of vector-borne bacterial diseases (*Anaplasma* spp. and *Ehrlichia canis*) in Spain and Portugal. BMC Vet Res. 2024;20(1):526. doi: 10.1186/s12917-024-04383-3 39593089 PMC11590303

[62] Tesla B, Demakovsky LR, Mordecai EA, Ryan SJ, Bonds MH, Ngonghala CN, et al. Temperature drives Zika virus transmission: evidence from empirical and mathematical models. Proc Biol Sci. 2018;285(1884):20180795. doi: 10.1098/rspb.2018.0795 30111605 PMC6111177

[63] Vicco A, McCormack C, Pedrique B, Ribeiro I, Malavige GN, Dorigatti I. A scoping literature review of global dengue age-stratified seroprevalence data: estimating dengue force of infection in endemic countries. eBioMedicine. 2024;104:105134. doi: 10.1016/j.ebiom.2024.105134 38718682 PMC11096825

[64] Pigott DM, Millear AI, Earl L, Morozoff C, Han BA, Shearer FM, et al. Updates to the zoonotic niche map of Ebola virus disease in Africa. eLife. 2016;5:e16412. doi: 10.7554/eLife.16412 27414263 PMC4945152

[65] Glass GE, Ganser C, Kessler WH. Validating Species Distribution Models With Standardized Surveys for Ixodid Ticks in Mainland Florida. J Med Entomol. 2021;58(3):1345–51. doi: 10.1093/jme/tjaa282 33386731 PMC8122235

[66] Whelan MG, Ware H, Ranka H, Kenny S, Shaikh S, Roell Y, et al. ArboTracker: a multipathogen dashboard and data platform for arbovirus seroprevalence studies. Lancet Infect Dis. 2024;24(11):e670–1. doi: 10.1016/s1473-3099(24)00585-1 39270692

[67] Gaythorpe KA, Hamlet A, Jean K, Garkauskas Ramos D, Cibrelus L, Garske T, et al. The global burden of yellow fever. eLife. 2021;10:e64670. doi: 10.7554/eLife.64670 33722340 PMC7963473

[68] Shearer FM, Longbottom J, Browne AJ, Pigott DM, Brady OJ, Kraemer MUG, et al. Existing and potential infection risk zones of yellow fever worldwide: a modelling analysis. Lancet Global Health. 2018;6(3):e270–8. doi: 10.1016/S2214-109X(18)30024-X 29398634 PMC5809716

